# Obstetrics

**Published:** 1894-06

**Authors:** L. M. Griffiths


### OBSTETRICS.

Transcendental philosophers who are fond of maintaining that parturition and the processes which lead up to it are purely physiological must often find it difficult to explain the complications of pregnancy. These may in the language of the text-books be pathological exaggerations of physiological processes, but in too many instances the pathology has so overshadowed the physiology that we are face to face with veritable diseases.

Recent communications have directed fresh attention to some of these. Among the most valuable of these contributions to periodical literature is that of Dr. Edward P. Davis on "Toxæmia of Pregnancy."[2] He says that the failure of the kidneys to excrete the additional quantity of waste products in the pregnant woman is not in itself sufficient to explain the condition in which the evidence points strongly to an acute intoxication with the products of bacteria. This condition is not of necessity evidenced by the presence of albumen in the urine. Dr. Davis lays special stress on the importance of carefully estimating the amount of urine secreted, and on the value of microscopic examination of urinary sediments. If there are present symptoms of toxæmia, such as a gradual diminution in the excretions, both solid and liquid; loss of appetite, with complaint of slight nausea or gastric distress; headache, a clammy skin, or, in some instances, a dry skin with deficient perspiration, and lassitude, mental and physical; and if it is found that the percentage of urea has fallen below 1.5, the excretory processes should be stimulated by promoting the action of the kidney, liver, intestine, skin, and lungs. Examination of the urine in 84 cases was frequently made before and after parturition. Marked diminution in the quantity of urea occurred only in cases having, or threatened with, eclampsia or manifesting symptoms of marked toxæmia. The diagnosis of toxæmia is greatly aided by attention to the patient's nervous system, which is not in a condition of simple apprehension but of intoxication varying in degree. Toxæmia may be present even when the examination of the urine fails to reveal either casts, albumen, or marked deficiency in urea.

For treatment, calomel and soda, not potash, should be occasionally given, and also purgatives such as colocynth, which produce free and liquid stools. A hot or warm bath should be taken at night, and the patient should drink a small quantity of warm water. Sedatives should be avoided. The patient's need is for elimination, and that should be prompt and thorough. If eclampsia threaten, the uterus should be at once emptied under chloroform.

Not the least of the troubles of pregnancy is persistent vomiting, and although happily the cases are rare in which labour has to be induced on account of this condition, much distress is occasioned by this complication; and if any recent additions to the therapeutic armamentarium can be employed for its relief, due prominence should be given to them.

The Hon. Dr. Creed reports[1] the case of Mrs. B., aged 22, whom he had seen in consultation. Dr. Jamieson, the ordinary attendant, had been requested to see her when she was nearly eight months pregnant with her third child. She was vomiting almost everything she took. The usual drugs recommended for the purpose of checking vomiting had no effect. In the course of a few days she gave birth to an eight months' child that had been dead some days. The vomiting persisted, and six days after the premature labour Dr. Creed saw her. She was lying with a towel placed under her mouth, having become too weak to be able to use a basin into which to vomit. She was retching incessantly. The tongue was very red and dry, the lips cracked, the pulse rapid and weak, and she appeared to be almost moribund. Dr. Creed hypnotised her, and made suggestions that the retching should cease and that she would not feel any further nausea, but have a desire for food. Immediately after she said she felt better. She did not vomit during the next three days. But on the next day she vomited twice after taking champagne. Suggestions whilst she was hypnotised were again successful. From the time of the first hypnosis the patient began to improve, and about ten days after was up and about. Hypnotism, as a therapeutic measure having had good results, should be tried in any case of obstinate vomiting during pregnancy before operative proceedings are undertaken.

Dr. Clara T. Dercum divides into two classes the various theories that have been proposed in regard to the etiology of hyperemesis gravidarum. In the first class she places all those cases in which are present any local lesions, whether uterine or gastro-intestinal. In the second class occurring in women of a neurotic type are the cases which present no local lesion, and which are the most serious in their nature. When the vomiting is accompanied with nausea and ptyalism, showing that the higher nervous centres are involved, the condition may at any

[1] *Australas. M. Gaz.*, vol. xii., 1893, p. 370.

time become grave. Dr. Dercum records a case that came under her own observation which presented unusual features. A neurotic woman, pregnant for the third time, had vomiting, nausea, and ptyalism commencing about a fortnight after conception and continuing for about four weeks, at the end of which period the symptoms were present night and day, and independent of food or drink. The prostration was extreme. The question of inducing abortion was considered, but instead it was determined to try feeding by the bowel, combined with rectal and hypodermatic medication. In three days her condition had improved, and six days later the patient suddenly ceased vomiting after eating a lamb chop. The record does not say that it was on account of this. After five days, all medicine and food was taken by the mouth and retained. But in little more than a fortnight the bad symptoms returned, and the general condition again became alarming. As there was no improvement with the same kind of treatment as before, abortion was induced at about the twelfth week from the beginning of pregnancy. Twins were delivered: the placental tissue was adherent. The vomiting ceased immediately after the expulsion of the contents of the uterus, but recovery was complicated by inflammatory pelvic troubles, and the patient was not well for a couple of months.

Dr. Dercum seeks to explain the subsidence of the symptoms in the course of the pregnancy by the fact that as little nourishment was taken for some time the fœtuses developed very slowly, and thus the cause of the nervous manifestations remained in abeyance. But this theory can not be sound, as the serious symptoms were developed in the early part of the pregnancy.

Dr. Boardman Reed also relates[1] a case in which serious vomiting was present, and subsided only to return with greater force. When the cervix was being dilated, in the hope that this would stop the vomiting, labour came on and a three months' fœtus was expelled. The patient sank into a condition of extreme exhaustion with high fever and died. Dr. Reed says that the life of a wife and mother is infinitely more important than that of an unborn babe, even when known to be alive and near full term, and to risk such a life out of consideration for a fœtus ought not to be thought of for a moment.

Dr. John G. Cecil refers[2] to two recent deaths from this cause, and records a case which he himself had seen in consultation. The patient had persistent vomiting from the time of her fourth conception. When first seen by Dr. Cecil she was in the third month of her pregnancy. The usual remedies had been given unsuccessfully. The uterus was retroverted and tender. As it was thought that the displacement was the

1 *Med. News*, vol. lxiv., 1894, p. 47.

2 *Med. Age*, vol. xii., 1894, p. 234.

cause of the vomiting, efforts were made to keep the uterus in position, but these were of no avail. The general condition got worse. An attempt to induce abortion failed, and the patient died undelivered. Dr. Cecil says that this case is one more confirmation of the conclusion of those who consider that patients often die from this complication because the abortion has not been induced early enough. The exact time when this should be done must always be a matter for serious consideration. Severe and protracted vomiting often ceases spontaneously or in reply to appropriate treatment; but no case should be allowed to approach a typhoid state, when interference would be too late.

The last Report of the Rotunda Hospital records[1] a death from excessive vomiting in pregnancy. The patient, who was six months pregnant with her first child, was admitted in a collapsed state. She had been vomiting for more than a month. All medicines had been without avail. She died undelivered two days after admission.

L. M. GRIFFITHS.

